# Identification of Genes and miRNAs Associated with TAFI-Related Thrombosis: An in Silico Study

**DOI:** 10.3390/biom13091318

**Published:** 2023-08-28

**Authors:** Erasmia Rouka, Sotirios G. Zarogiannis, Chrissi Hatzoglou, Konstantinos I. Gourgoulianis, Foteini Malli

**Affiliations:** 1 Department of Nursing, School of Health Sciences, University of Thessaly, GAIOPOLIS, 41500 Larissa, Greece; 2 Department of Physiology, Faculty of Medicine, School of Health Sciences, University of Thessaly, BIOPOLIS, 41500 Larissa, Greece; szarog@uth.gr (S.G.Z.); chatz@uth.gr (C.H.); 3 Department of Respiratory Medicine, Faculty of Medicine, School of Health Sciences, University of Thessaly, BIOPOLIS, 41500 Larissa, Greece; kgourg@uth.gr

**Keywords:** coagulation, fibrinolysis, hemostasis, interactome, miRNAs, TAFI, Thrombin-Activatable Fibrinolysis Inhibitor, thrombosis, transcription factors

## Abstract

Thrombin-Activatable Fibrinolysis Inhibitor (TAFI) is a carboxypeptidase B-like proenzyme encoded by the *CPB2* gene. After thrombin activation, TAFI downregulates fibrinolysis, thus linking the latter with coagulation. TAFI has been shown to play a role in venous and arterial thrombotic diseases, yet, data regarding the molecular mechanisms underlying its function have been conflicting. In this study, we focused on the prediction and functional enrichment analysis (FEA) of the TAFI interaction network and the microRNAs (miRNAs) targeting the members of this network in an attempt to identify novel components and pathways of TAFI-related thrombosis. To this end, we used nine bioinformatics software tools. We found that the TAFI interactome consists of 28 unique genes mainly involved in hemostasis. Twenty-four miRNAs were predicted to target these genes. Co-annotation analysis of the predicted interactors with respect to Kyoto Encyclopedia of Genes and Genomes (KEGG) pathways and transcription factors (TFs) pointed to the complement and coagulation cascades as well as neutrophil extracellular trap formation. Cancer, stroke, and intracranial aneurysm were among the top 20 significant diseases related to the identified miRNAs. We reason that the predicted biomolecules should be further studied in the context of TAFI-related thrombosis.

## 1. Introduction

The hemostatic process has been defined as “a host defense mechanism to preserve the integrity of the closed high pressure circulatory system” [1]. Balanced hemostasis is maintained by the coordination of the coagulation and fibrinolytic systems [2]. Similar to the coagulation cascade, fibrinolysis is highly and tightly regulated by several molecules such as cofactors, inhibitors, and receptors [2]. Thrombin-Activatable Fibrinolysis Inhibitor (TAFI) is a 55-kDa carboxypeptidase B-like proenzyme that is encoded by the *CPB2* gene [3]. Eaton et al. were the first to report that TAFI is synthesized in the liver as a preproenzyme that is secreted into the plasma as a proenzyme, after the removal of the 22-residue signal peptide [4,5]. The presence of TAFI in platelets was established in 2003 [6]. Following thrombin activation, TAFI downregulates fibrinolysis, thus linking the latter with coagulation [5]. Two pools of TAFI are present in blood, the plasma pool and the platelet pool, which is only secreted upon platelet activation and accounts for less than 0.1% of total blood TAFI [7,8].

Activated TAFI (TAFIa) attenuates the fibrinolytic response by removing the C-terminal lysines from the fibrin surface thus decreasing its cofactor activity [5,8]. This function of TAFIa is exerted through a threshold-dependent mechanism which is correlated to the concentrations of the tissue-type plasminogen activator (tPA), α2-antiplasmin and plasmin [5]. Thrombin and plasmin are significant TAFI activators, yet, the rate of activation is accelerated by the thrombin-thrombomodulin complex, which cleaves TAFI with a significantly higher catalytic efficiency compared with thrombin alone [8]. The primary regulatory mechanism of TAFIa involves its intrinsic thermal instability, which has been extensively reviewed [5,8]. The rate of TAFI activation and the stability of TAFIa function to maintain its concentration above the threshold value required for fibrinolysis suppression [8]. Consequently, the extent of the effect of TAFIa on fibrinolysis but also inflammation—for which a potentially protective role of TAFIa has been suggested in recent years—reflects the thermal stability of the enzyme [8].

Due to its role as the molecular hub of clotting and thrombolysis, TAFI has been associated with the pathophysiology of venous and arterial thrombotic diseases, as these are often caused by either an increased coagulation or an impaired fibrinolytic response [5]. Acquired disorders of the fibrinolytic components such as hyperfibrinolysis and hypofibrinolysis, resulting in bleeding and thrombosis, respectively, have been described in several diseases [2].

Existing evidence has supported the link between elevated TAFI levels and venous thromboembolic disease, deep vein thrombosis (DVT), stroke, and coronary heart disease [5]. The AtheroGene study also found that the amount of TAFIa is independently associated with the risk of death from cardiovascular causes [9]. Notably, in the context of acute pulmonary embolism (PE), low TAFI concentrations and low alpha-2-antiplasmin correlated with worse PE severity [7]. Yet, other studies have reported opposing findings as to TAFI levels in the aforementioned disease categories. For example, decreased plasma levels of total TAFI have been observed in angiographically documented coronary artery disease patients presenting with stenosis above 70% [10]. Another study investigating TAFI activity and antigen plasma levels in patients admitted to a coronary care unit found no differences in TAFI levels, either activity or antigen, between patients and healthy controls matched for age and sex [11]. A case-control study assessing TAFI antigen levels with regard to markers of coagulation and fibrinolysis in patients with and without acute PE found no association between TAFI antigen levels and the presence of acute PE [12]. The same study reported a relation of TAFI antigen levels with pulmonary occlusion rate and suggested that in subjects with massive PE, plasma TAFI levels might be secondarily increased during blood coagulation due to the release from activated platelets [12]. Data have also been controversial as to the contribution of the TAFI gene polymorphism towards the development of cardiovascular disease [5]. In addition, there is inconsistency across studies results with respect to the function of TAFI in murine models of thrombosis [5,8]. Significant differences in the models used to induce thrombosis in these studies as to the localization and magnitude of the thrombogenic stimulus have been thought to explain this discrepancy [8].

Research in the field of hemostasis and thrombosis is of crucial importance in relation to the development of new therapies for pathologies such as DVT, stroke, PE, and hemorrhaging related diseases [13], as these contribute significantly to the global burden of disease (GBD) [14]. In the GBD 2019 study, ischemic heart disease and stroke were reported as the top-ranked causes of disability-adjusted-life-years (DALYs) in both the 50–74-years and 75-years and older age groups [14]. Computational models and methods complementing experimental studies can enrich our understanding of the molecular mechanisms by which such diseases develop and progress [13], thus having a significant role in drug discovery. Of note, due to its anti-fibrinolytic effect and association with risk for thromboembolic disorders, TAFIa continues to be a putative drug target [5]. Pharmacological strategies including synthetic peptides, small molecule inhibitors, monoclonal antibodies, and nanobodies that target TAFI or TAFIa have been thoroughly reviewed [5]. Existing evidence has highlighted the crucial role of computational modeling for the field of thrombosis and hemostasis, as our knowledge on pathological states associated with thrombi formation can be increased using various computational methods [13]. In the field of bioinformatics, enrichment analysis of genes, transcriptional and post-transcriptional regulators has shown great potential for the discovery of significant biological functions related to research questions under investigation. To our knowledge, only one in silico study has determined the structural and functional impact of TAFI and its expression [15].

In view of the above mentioned and considering the inconsistency between reported data on TAFI-mediated thrombotic processes, in this study we used an in silico approach towards the prediction and functional enrichment analysis (FEA) of the TAFI interaction network and the microRNAs (miRNAs) targeting this network so as to identify novel components of TAFI-related thrombosis and to explore their possible involvement in the pathophysiology of clotting disorders.

## 2. Materials and Methods

### 2.1. Identification of the TAFI Interactome and Associated miRNAs

An analysis in three independent databases (STRING v.11.5; http://string-db.org/, GeneMANIA; http://www.genemania.org/), and BioGRID v. 4.4; http://thebiogrid.org/) [16,17,18] was performed to combine data from multiple bioinformatic resources, thus obtaining the TAFI interactome with the best possible accuracy. The STRING database integrates physical and functional protein–protein interactions [16]. GeneMANIA combines functional association data including protein and genetic interactions, pathways, co-expression, co-localization, and protein domain similarity [17]. BioGRID consolidates genetic and protein interaction data curated from both high-throughput datasets and individual focused studies [18]. Gene names of the predicted interactors were retrieved from GeneCards, the Human Gene compendium which integrates gene-centric data from approximately 150 web sources, including genomic, transcriptomic, proteomic, genetic, clinical, and functional information [19]. The software tool FunRich v3.1.3. (Melbourne, Australia) was accessed for the prediction of the miRNAs that target the identified TAFI functional partners (http://www.funrich.org/) [20]. Analyses were performed in January 2023 using the default parameters.

### 2.2. FEA of the Predicted TAFI Interactors and Associated miRNAs

FEA of gene ontology (GO) annotations relative to Biological Processes and Tissue Expression of the TAFI interactors was performed using the Database for Annotation, Visualization and Integrated Discovery (DAVID) which consists of a comprehensive knowledgebase and a set of functional analysis tools [21]. The functional enrichment and interaction network analysis software FunRich v3.1.3 (Melbourne, Australia) [20] was used to identify significantly enriched GOs of Biological Pathways (BPs) and Transcription Factors (TFs) for both the TAFI interactome and associated miRNAs. A co-annotation analysis of the predicted interactors with respect to functional (Kyoto Encyclopedia of Genes and Genomes; KEGG pathways) and regulatory elements (TFs) was subsequently performed using the web server application GeneCodis 4 (https://genecodis.genyo.es) which integrates results from different omics and knowledge perspectives providing advanced gene set analysis of small RNAs, TFs and methylation sites [22]. Analyses were performed in February and August 2023.

### 2.3. RNA Disease Analysis of the Predicted miRNAs

The disease enrichment tool of the RNADisease v4.0 repository was employed to analyze diseases at the miRNA level (http://www.rnadisease.org/enrichment) [23]. RNADisease v4.0 provides a comprehensive and concise data resource of RNA-disease associations containing more than three million RNA-disease association entries. Default parameters were used, and the analysis was performed in February 2023.

### 2.4. Tissue Expression Exploration of the Predicted miRNAs and TFs

DIANA-miTED was used for the investigation of miRNA expression and distribution across blood, liver and bone marrow (https://dianalab.e-ce.uth.gr/mited/#/) [24]. The database contains expression values derived from more than 15,000 analyzed small RNA sequencing human datasets that were collected from the Sequence Read Archive (SRA) and The Cancer Genome Atlas (TCGA) [24]. Results were filtered to include healthy samples and expression was retrieved in log2 reads per million mapped reads (RPM) values. The EMBL-EBI Expression Atlas (https://www.ebi.ac.uk/gxa/home) [25] was queried for data from the Human Protein Atlas project [26] relative to the expression of the predicted TFs in the same tissues. Expression Atlas integrates data from more than 4500 expression studies from more than 65 different species, across different conditions and tissues [25]. Protein abundance values were retrieved as parts per billion. Analyses were performed in August 2023.

## 3. Results

### 3.1. Predicted Interactors and Associated miRNAs of the CPB2 Gene Encoding the TAFI Proenzyme

**Figure 1:**
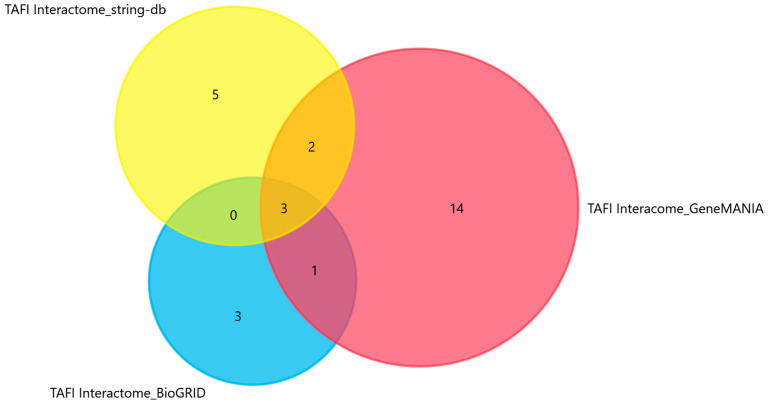
Venn diagram depicting the overlapping results among the three databases that were used in this study for the prediction of the TAFI interaction network.

Twenty-eight unique genes mainly involved in hemostasis and the clotting cascade were identified as functional partners of TAFI (Table 1). An illustration of the intersections between the STRING, BioGRID, and GeneMANIA results is presented in Figure 1. Twenty-four miRNAs were predicted to target the identified interactors (Table 2).

### 3.2. FEA Results for the Predicted TAFI Interactors and Associated miRNAs

#### 3.2.1. DAVID FEA Results for the TAFI Interactome in Relation to Biological Processes and Tissue Expression

With respect to biological processes, the most significantly enriched GO terms were hemostasis and blood coagulation (Table 3). The annotations with the higher level of confidence concerning tissue expression were plasma, cerebrospinal fluid, liver, platelet, and serum (Table 4).

#### 3.2.2. FunRich FEA Results for the TAFI Interactors and Associated miRNAs with Respect to BPs and TFs

**Figure 2:**
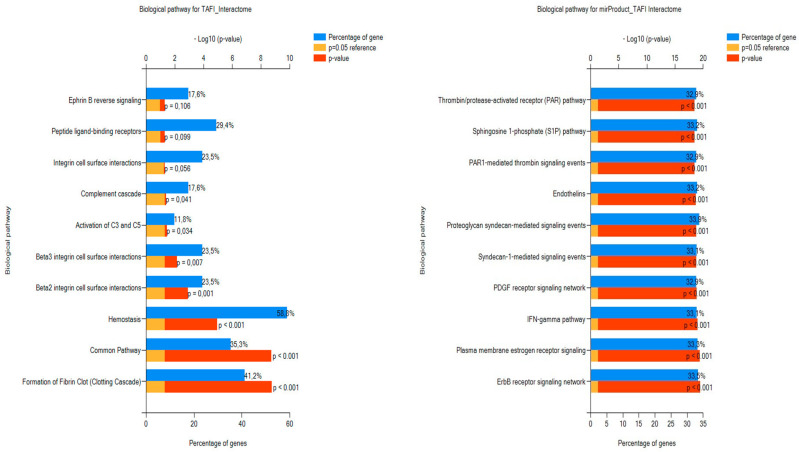
The top ten ranked annotations for BP GOs of the TAFI interactors and associated mi-RNAs as retrieved by FunRich.

**Figure 3:**
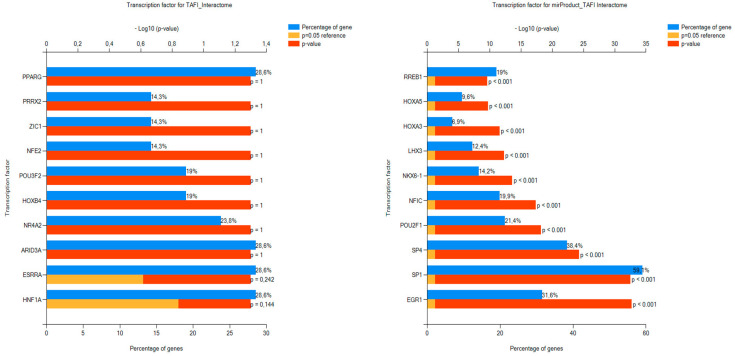
The top ten ranked annotations for TF GOs of the TAFI interactors and associated mi-RNAs as retrieved by FunRich.

**Figure 4:**
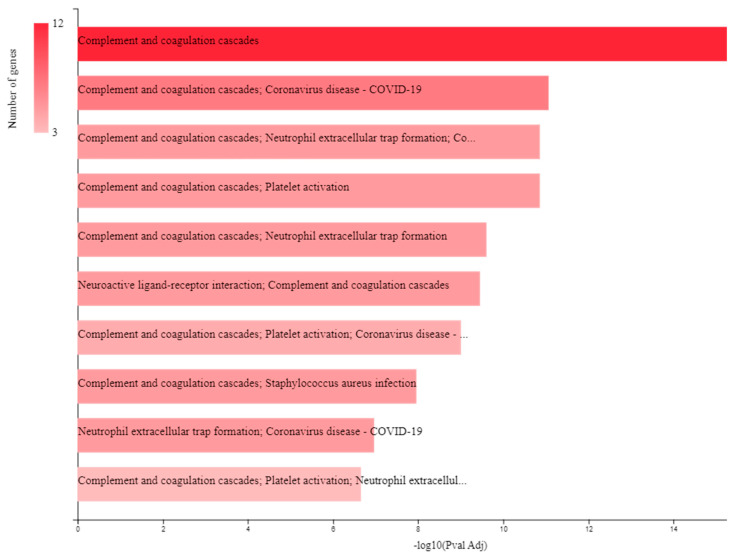
Results of the GeneCodis co-annotation analysis of the predicted TAFI interactors with respect to KEGG pathways and TFs.

Regarding BPs, the function where the input TAFI interactors were over-represented was hemostasis followed by the clotting cascade. FEA relative to BPs of the miRNAs targeting the TAFI interactome verified that the predicted RNA molecules are mainly involved in the regulation of coagulation and thrombosis (Figure 2). In the context of TF GOs for miRNAs, the top ten significantly enriched annotations were the regulatory molecules RREB1, HOXA5, HOXA3, LHX3, NKX6-1, NFIC, POU2F1, SP4, SP1, and EGR1. No significant enrichment was found for biomolecules regulating the transcription rate of TAFI interactors (Figure 3), yet, co-annotation analysis of the predicted interactome with respect to KEGG pathways and TFs pointed to the complement and coagulation cascades as well as neutrophil extracellular traps (NETs) formation (Figure 4).

### 3.3. Diseases Associated with the miRNAs Targeting the TAFI Interactome

**Figure 5:**
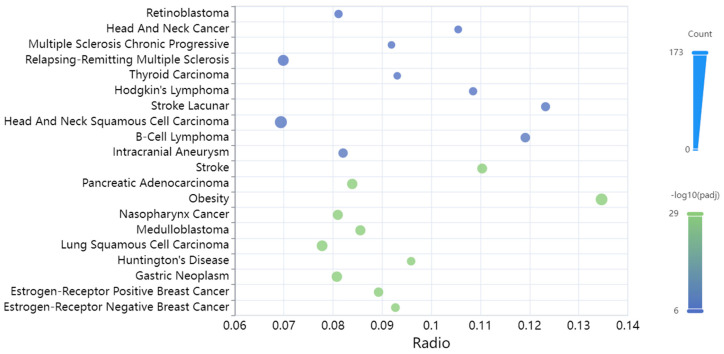
The 20 most significantly enriched diseases for the TAFI interactome-associated miRNAs.

The diseases where the input miRNAs were over-represented were cancer along with multiple sclerosis, obesity, Huntington’s disease, stroke, and intracranial aneurysm (Figure 5).

### 3.4. Expression Values of the Query miRNAs and TFs in Blood, Liver and Bone Marrow

**Figure 6:**
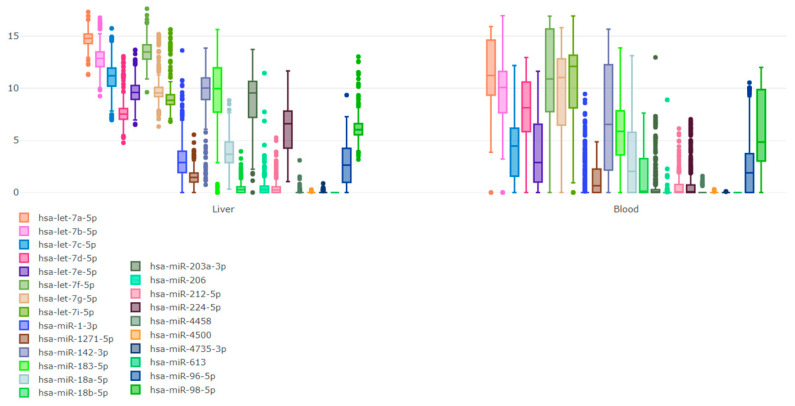
Abundance distribution per tissue of interest of the predicted miRNAs as retrieved by the DIANA-miTED database. No results were available for bone marrow.

**Figure 7:**
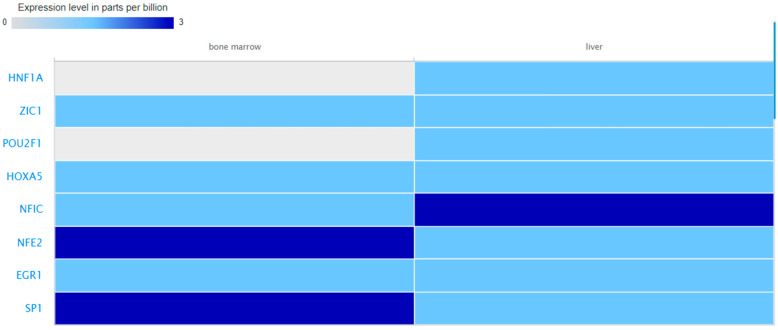
Expression levels of the HNF1A, ZIC1, POU2F1, HOXA5, NFIC, NFE2, EGR1, and SP1 TFs in the selected tissues as retrieved by the EMBL-EBI Expression Atlas. No data were available for the remaining TFs and for blood.

Boxplots showing log2RPM expression values of the predicted miRNAs in liver and blood are presented in Figure 6. Proteomics data from bone marrow and liver were retrieved for eight TFs. The expression level of theses biomolecules is depicted in Figure 7.

## 4. Discussion

Considering the scarce and often conflicting results with respect to the contribution of TAFI in the pathophysiology of venous and arterial thrombotic disorders, this study aimed to explore the potential relevance of TAFI-related genes and miRNAs to thrombosis. Twenty-eight unique interactors were identified as functional partners of the *CPB2* gene, which encodes the TAFI zymogen, and twenty-four miRNAs were predicted to mediate the posttranscriptional silencing of the identified interactome. With respect to Biological Processes GOs, TAFI interactors were over-represented in hemostasis and blood coagulation. In terms of Tissue Expression GOs, 15 genes were correlated to the liver annotation, while 12 and 5 genes were over-represented in the plasma and platelets annotation, respectively. These findings enhanced confidence in the in silico prediction of molecular interactions between TAFI and the identified genes.

Pathway enrichment analysis of the determined biomolecules pointed to signaling cascades associated with thrombotic processes. As to the TAFI interaction network, hemostasis, fibrin clot formation, common pathways, beta 2/beta 3 integrin cell surface interactions, and C3/C5 complement activation were the most significantly enriched annotations in the context of BP GOs. Integrin ligand interactions have been shown to play important roles in angiogenic, thrombotic, infectious, and inflammatory processes [27]. Integrins of the Beta 2 subgroup (β2) are considered key regulators of adhesion, leukocyte recruitment, and immune cell signaling [27]. The receptor protein alphaIIb/beta3 (αIIbβ3) participates in thrombosis and fibrosis by promoting platelet adhesion and aggregation via coupling with soluble fibrinogen, fibronectin, and the von Willebrand factor (VWF) [27]. Activation of complement components such as C3 and C5 has also been linked to platelet-mediated thrombosis with implications for disease development including, but not limited to, myocardial infarction, stroke, PE, and venous thromboembolism [28].

Ranked retrieval annotations for BP GOs of the miRNAs that target TAFI interactors involved signaling networks which have been associated with coagulation and/or inflammation such as the protease-activated receptor (PAR) [29], sphingosine-1-phosphate (S1P) [30], syndecan-1 (SDC-1) [31], platelet-derived growth factor (PDGF) [32], interferon γ (IFNγ) [33], estrogen, and ErbB receptor pathways [34,35].

With respect to the FunRich FEA results for TF GOs of the TAFI interactors and associated miRNAs, only PPARG was recently reported to play contradictory roles in the pathophysiology of thrombosis and atherosclerosis [36]. Hence, the remaining TFs identified in this study might be biomolecules with potential relevance to mechanisms of thrombus formation. This hypothesis is further supported by the GeneCodis co-occurrence discovery of BP and TF GOs, which pointed to the complement and coagulation cascades as well as the formation of NETs. A growing body of research has suggested the contribution of the latter extracellular structures in the abnormal activation of the coagulation pathway and the emergence of pathological thrombi [37]. Of interest, NK cell-dependent IFN-γ production has been shown to promote venous thrombosis through the formation of NETs by neutrophils [33]. In the context of COVID-19 disease, NETs have been reported as the most important underlying factor for the increased thrombotic events, vascular injuries, and organ damage occurring in affected patients [38].

Another important finding of this study is that only 11 (hsa-miR-96-5p, members of the let-7 family, and hsa-miR-183-5p) out of the 24 miRNAs which were predicted to target the TAFI interactome have been functionally validated in relation to the post-transcriptional control of genes involved in hemostasis and thrombo-inflammation [39]. Of note, it was previously reported that the let-7 miRNA family represents 48% of the platelet miRNA content [40]. In terms of gene expression regulators, it should be emphasized that the same TF can regulate different genes in different cell types and that tissue- and cell-type-specific expression of TFs may be suggestive of specific functions [41]. For example, HNF1, one of the first characterized liver enriched TFs [42] has been shown to play a role in the hepatic expression of the Insulin-like growth factor binding protein-1 (IGFBP-1) [43,44]. NFE2 has been reported as an essential factor for megakaryocyte maturation and platelet formation regulating critical target genes independent of the action of thrombopoietin [45]. Similarly, the expression patterns of miRNAs can be tissue- and cell-specific [46]. Considering that tissue-specific miRNAs and TFs may influence the expression profile and functional roles of TAFI-related genes, in this study, we also explored the abundance of the predicted transcriptional and post-transcriptional regulators in blood, liver, and bone marrow to allow for comparisons. Although we were not able to retrieve data for all relevant biomolecules and/or tissue environments, we have provided findings which confirm the presence of several of the predicted regulatory elements in the selected tissues and thus enhance the possibility of their involvement in the regulation of TAFI-related genes. These results may facilitate future experimental investigation in these distinct organismal parts.

Disease enrichment analysis of the identified miRNAs indicated their relation to several cancer types as well as stroke and intracranial aneurysm (IA). Available evidence regarding the effect of the coagulation cascade on cancer cells and the tumor microenvironment was recently revised, emphasizing the need for a simultaneous exploration of the dynamics of coagulation and fibrinolysis [47]. Stroke and IA share common risk factors such as female sex, hypertension, and large vessel occlusion [48]. The two conditions are thought to be associated to some extent, as the prevalence of unruptured IA is notably higher in ischemic stroke patients as compared to the general population [48]. Five major phenotypes of stroke have been reported to occur through thrombogenic pathways reflecting the phenotypic complexity created by thrombosis/hemorrhage [49]. Of note, POU3F2, a TF associated with the predicted TAFI interactome has been reported to be expressed almost exclusively in the cerebral cortex [41].

Matrix metalloproteinase-10 (MMP-10) and TAFI have been shown to colocalize with inflammatory cells and platelets in all thrombi retrieved from large vessel occlusion stroke patients [50]. Most importantly, subjects who died within 3 months had higher VWF and lower TAFI content in thrombi, suggesting that the histological composition and distribution of different thrombi hemostatic components have prognostic and therapeutic implications [50]. Two recent reviews have highlighted the impact of thrombus composition and organization on the efficacy of thrombolysis in the setting of acute ischemic stroke (AIS) [51,52]. Potential thrombolytic therapies consist of targeting either the fibrin network or non-fibrin AIS thrombus components, such as VWF multimers, platelet aggregates, or extracellular DNA [52].

As regards to IAs, there has been an increased research interest towards the influence of miRNAs on their development and/or rupture, as this class of small non-coding RNAs has been shown to regulate the proliferation, migration, and apoptosis of vascular smooth muscle cells (VSMCs) [53]. Phenotypic modulation of VSMCs has been reported as one of the targeted therapies for IAs [53]. Jin et al. [54] investigated the level of serum miRNA-21 in patients with IA of different phases and found that the mean relative expression levels of this specific molecule in quantitative real time polymerase chain reaction were significantly lower in all experimental groups as compared to the angiography negative (control) group. Extracellular vesicles and their associated miRNAs have emerged as potential biomarkers for detecting IAs early, predicting rupture, and assessing the severity of the disease [55].

Various miRNAs have also been suggested to play important roles in PE as biomarkers for its prediction in multiple pathologic events but also as potential novel therapeutic targets [56]. In the context of clinical applications, the combination of miRNA biomarkers and/or miRNA microchips with commonly used biomarkers for thrombotic diseases such as D-dimer has been proposed as a promising predictive tool for PE occurrence and prognosis [56]. In addition, it has been reported that the implementation of machine learning approaches will be a requisite in the future for establishing the sensitivity of miRNAs and their clinical relevance in acute PE, which represents the most serious complication of venous thromboembolism, thus necessitating early diagnosis and treatment [57]. These reports denote the importance of investigating miRNA-mediated gene expression regulation in the context of hemostasis and thrombosis.

## 5. Conclusions

Taken together, our data indicate that the identified miRNAs and TFs have a role in controlling the activity of genes associated with TAFI-related thrombosis. Most importantly, several of these biomolecules have yet to be investigated within the context of bone marrow megakaryocytes, platelets, and hepatocytes transcriptional profiling. A limitation of this study is that it relies on the implementation of in silico methodology. Hence, wet lab research is required to corroborate the bioinformatics predictions. Yet, our findings provide the basis for evidence-directed validation experiments of the TAFI-interactome and associated gene expression regulators in the setting of thrombosis.

## Figures and Tables

**Table 1:** The predicted by STRING, BioGRID and GeneMANIA *CPB2* interactome.

Gene Symbol	Gene Name as Retrieved by GeneCards
*F2*	Coagulation Factor II, Thrombin
*THBD*	Thrombomodulin
*PLG*	Plasminogen
*C5*	Complement C5
*C3*	Complement C3
*F2R*	Coagulation Factor II Thrombin Receptor
*SERPINC1*	Serpin Family C Member 1
*AGT*	Angiotensinogen
*UBE2A*	Ubiquitin Conjugating Enzyme E2 A
*FGA*	Fibrinogen Alpha Chain
*A2M*	Alpha-2-Macroglobulin
*ERO1LB*	Endoplasmic Reticulum Oxidoreductase 1 Beta
*FN1*	Fibronectin 1
*TINAG*	Tubulointerstitial Nephritis Antigen
*FGG*	Fibrinogen Gamma Chain
*FGB*	Fibrinogen Beta Chain
*PITRM1*	Pitrilysin Metallopeptidase 1
*CPA1*	Carboxypeptidase A1
*CPA6*	Carboxypeptidase A6
*CPA5*	Carboxypeptidase A5
*CPA4*	Carboxypeptidase A4
*CPB1*	Carboxypeptidase B1
*ACY1*	Aminoacylase 1
*CPA2*	Carboxypeptidase A2
*CPA3*	Carboxypeptidase A3
*MBL2*	Mannose Binding Lectin 2
*CP*	Ceruloplasmin
*CYP2C8*	Cytochrome P450 Family 2 Subfamily C Member 8

**Table 2:** The miRNAs that were predicted to target the *CPB2* gene interactors as retrieved by the FunRich software.

miRNAs	RNA ID
hsa-miR-18a-5p	MIMAT0000072
hsa-miR-142-3p	MIMAT0000434
hsa-miR-18b-5p	MIMAT0001412
hsa-miR-4735-3p	MIMAT0019861
hsa-miR-96-5p	MIMAT0000095
hsa-miR-1-3p	MIMAT0000416
hsa-miR-206	MIMAT0000462
hsa-miR-613	MIMAT0003281
hsa-miR-1271-5p	MIMAT0005796
hsa-miR-203a-3p	MIMAT0000264
hsa-miR-224-5p	MIMAT0000281
hsa-let-7a-5p	MIMAT0000062
hsa-let-7b-5p	MIMAT0000063
hsa-let-7c-5p	MIMAT0000064
hsa-let-7d-5p	MIMAT0000065
hsa-let-7e-5p	MIMAT0000066
hsa-let-7f-5p	MIMAT0000067
hsa-miR-98-5p	MIMAT0000096
hsa-let-7g-5p	MIMAT0000414
hsa-let-7i-5p	MIMAT0000415
hsa-miR-4458	MIMAT0018980
hsa-miR-4500	MIMAT0019036
hsa-miR-183-5p	MIMAT0000261
hsa-miR-212-5p	MIMAT0022695

**Table 3:** Ranked annotations for Biological Processes GOs of the TAFI interactors as retrieved by the DAVID Functional Annotation tool.

Term	Gene Count	*p*-Value	Benjamini
Hemostasis	8	1.2E−13	1.5E−12
Blood coagulation	8	1.2E−13	1.5E−12
Complement pathway	3	9.1E−4	7.6E−3
Innate immunity	5	1.7E−3	1.1E−2
Complement alternate pathway	2	1.6E−2	7.9E−2
Acute phase	2	2.6E−2	1.0E−1
Immunity	5	2.9E−2	1.0E−1

**Table 4:** Ranked annotations for GOs relative to Tissue Expression of the TAFI interactors as retrieved by the DAVID Functional Annotation tool.

Term	Gene Count	*p*-Value	Benjamini
Plasma	12	7.4E−14	4.1E−12
Cerebrospinal fluid	3	1.9E−3	5.4E−2
Liver	15	4.8E−3	8.4E−2
Platelet	5	6.0E−3	8.4E−2
Serum	2	2.9E−2	2.9E−1
Bile	2	3.1E−2	2.9E−1
Urine	2	4.0E−2	3.2E−1
Milk	2	4.6E−2	3.2E−1
Blood	4	8.6E−2	5.3E−1

## Data Availability

The data used to support the findings of this study are included within the article.
